# *P* in the right place: Revisiting the evidential value of *P*‐values

**DOI:** 10.1111/jebm.12319

**Published:** 2018-11-05

**Authors:** Per Lytsy

**Affiliations:** ^1^ Department of Public Health and Caring Sciences Uppsala University Uppsala Sweden; ^2^ Department of Clinical Neuroscience Karolinska Institute Stockholm Sweden

**Keywords:** evidence, hypothesis, *P*‐value, replication, statistics

## Abstract

*P*‐values are often calculated when testing hypotheses in quantitative settings, and low *P*‐values are typically used as evidential measures to support research findings in published medical research. This article reviews old and new arguments questioning the evidential value of *P*‐values. Critiques of the *P*‐value include that it is confounded, fickle, and overestimates the evidence against the null. *P*‐values may turn out falsely low in studies due to random or systematic errors. Even correctly low *P*‐values do not logically provide support to any hypothesis. Recent studies show low replication rates of significant findings, questioning the dependability of published low *P*‐values. *P*‐values are poor indicators in support of scientific propositions. *P*‐values must be inferred by a thorough understanding of the study's question, design, and conduct. Null hypothesis significance testing will likely remain an important method in quantitative analysis but may be complemented with other statistical techniques that more straightforwardly address the size and precision of an effect or the plausibility that a hypothesis is true.

## 1 INTRODUCTION

The *P*‐value is the most well‐known statistic, typically accompanying some measures of effect or association in scientific publications reporting the results of quantitative analyses. The *P*‐value is the result of a significance test; a test often credited to the statistical pioneer Ronald Fisher who published some seminal books and papers between 1920 and 1960 on the development of statistical methods. The *P*‐value quantifies the probability of obtaining data equal to or more extreme than the ones observed, given the assumption that the null hypothesis is true. Fisher regarded the *P*‐value as an informal but objective index of evidence against the null hypothesis, to be used by the researcher to judge whether data is compatible or not with the null.

Some years after Fisher introduced the significance test, two other statisticians, Jerzy Neyman and Egon Pearson, developed the theory of hypothesis testing, a test in which they let data determine if the null should be rejected or not in favor of an alternative hypothesis. Neyman and Pearson dismissed Fisher's evidential interpretation of *P*‐values and were more concerned about controlling long‐term error rates when performing hypothesis testing by reflected use of rejection levels and study power.

### 1.1 Evidence and decisions

Even though *P*‐values are not necessary for hypothesis testing strictly speaking (the null may be rejected if the test statistic falls within the rejection area), Fisher's test of significance and Neyman Pearson's rule of behavior were inevitably combined in the procedure known as null hypothesis significance testing. Most of today's medical journals reporting results from quantitative analyses lean on both Fisher's and Neyman Pearson's different schools of statistical inference. Authors are often asked to report exact *P*‐values (often together with 95 % confidence intervals of the estimates of effect sizes) as well as to perform hypothesis tests, that is, dichotomizing the main results into being statistically significant or not, given a preset level of rejection.

Much has been written about these different approaches, including that the hybrid method used today was not the intention by the founders.^1^, ^2^ Although some authors consider null hypothesis significance testing an optimal method for demonstrating evidence,^3^ others have pointed out that the method comes with logical flaws as well as interpretational difficulties.^4^, ^5^, ^6^, ^7^, ^8^ The *P*‐value is at the center of this debate, as the evidential implications of low *P*‐values have been shown to be unclear for students, academics, and even teachers of statistics.^9^, ^10^, ^11^ New theoretical developments and empirical findings cast additional doubt on the evidential value of *P*‐values, and the purpose of this article to present some old and new arguments.

### 1.2 Virtues and weaknesses of *P*

Before a criticism of the *P*‐value, some of its virtues need to be highlighted. *P*‐values are both well‐known and well‐established and have been called “the gold standard” of statistical validity.^12^ In the era of computers, *P*‐values are easily computed even for complicated statistics, and they provide estimates restricted between 0 and 1 with a fairly direct understanding; most schooled in statistics probably agree that a low *P*‐value may indicate something of interest, whereas this is less evident when the *P*‐value is high. Low *P*‐values are typically present in the results in clinical research publications reporting *P*‐values^13^; of value to researchers as they increase the chances of fast and successful publishing.^14^ Among the often repeated critiques of *P*‐values are that they are almost universally misinterpreted,^7^, ^15^ that they are fickle,^16^ and regularly overstate the evidence against the null.^17^, ^18^ *P*‐values have also been accused of being intrinsically deceptive as they confuse effect size and sample size into one confounded figure.^19^ Further allegations stress that *P*‐values are not objective measures^20^ and they do not bear the qualities we believe evidential measures should have, such as providing positive evidence, conveying only observed data, and providing an index that within itself compares two or more hypotheses.^6^ Nor do they, in fact, seem to logically qualify as being measures of support for or against anything.^21^

All these criticisms are worth reflection on their own merits. It is, however, possible to reduce them all to one question that is central to the purpose of a scientific investigation or experiment. When a researcher decides to test a hypothesis, he or she would like to know whether or not it is true. That question, however, is not possible to conclusively answer according to the Popperian view of science, as the absolute truthfulness of general hypotheses cannot be obtained using empirical techniques. Inferences from quantitative analyses, typically derived from population samples and statistical modeling, are, moreover, associated with uncertainty, making such methods even more ill‐suited for providing definite answers to general research questions. Results from statistical tests do, however, affect whether we hold a tested hypothesis as likely true or not. Assessing statistical test results is, then, a matter of judging their evidential support for or against a scientific proposition.

Unfortunately, the *P*‐value is not a reliable measure to use in that process. Many distinguished researchers and methodologists have tried to explain why.^5^, ^7^, ^8^, ^10^, ^12^ The brief story is this: frequentist statistics, the statistical framework to which null hypothesis significance testing and *P*‐values belong, cannot provide direct estimates of the truthfulness of hypotheses. Nor is a *P*‐value a trustworthy indicator of the truthfulness of a hypothesis, whether be it the null or the alternative.

Even if we follow a common advice, for example, the one provided by Sterne and Smith,^2^ and consider a very low *P*‐value (*P* < 0.001) as strong evidence against a null, it does not generally follow that the null hypothesis in such a test is likely false.^10^ There are several reasons for this. One is that the likeliness that the null is true (or false) may be more or less likely to start with. Hypotheses are not created equal. The hypothesis, which is tested within the model, has a targeted effect size, which is commonly set to zero effect. But the researcher may also decide to test other effect sizes as nulls, each being more or less likely to be true. The likeliness of the null being true is not assessed within the test but assumed to be true to enable a *P*‐value calculation.^8^

### 1.3 Not supportive

Furthermore, low *P*‐values only provide evidence that is against the null. Even if we rightfully reject a null hypothesis as false, this does not affirm the theory that led to the test.^7^ The *P*‐value says nothing about the alternative hypothesis. There are, moreover, typically additional and auxiliary hypotheses, other than the one proposed by the researcher, which may explain why a given set of data is unlikely under the null. A correctly rejected null gives no guidance which of the possible identified alternative hypotheses, if any, is correct.^8^ In fact, even an accurately low *P*‐value does not, logically, positively support any hypothesis. This is because *P*‐values only define evidence in relation to one hypothesis—the null.^6^

Despite the interpretational ambiguity of low *P*‐values, they are still, in some sense, viewed as supporting the research question. The rationale of such reasoning seems to be that even if low *P*‐values from seemingly well performed published research do not accurately depict truthfulness of scientific propositions every time, this is at least likely to be the case much of the time.

That reasoning has, however, been challenged. John Ioannidis claimed in a provocative article some years ago that most research findings are likely false.^22^ In the context of null hypothesis significance testing, a more specific question is this: we know that *P*‐values sometimes are falsely low, so how often do low *P*‐values and significant findings correctly refer something that is really true?

Unfortunately, we will never have a clear answer to that question. Since it is not possible with certainty to know whether a hypothesis with a general claim is true or not, there is, consequently, no clear standard to which low *P*‐values may be benchmarked as indicators of truth. But if we assume that null hypothesis significance testing, in general, is a practical method for separating false hypotheses from those that are true, and if we further assume that low *P*‐values in general often are likely to represent true findings, then low *P*‐values from well‐performed studies should typically turn out low also in study replications. Reproducibility is a key principle in science. A scientific claim is scientific only because it is supported by evidence. If the supporting evidence cannot be presented or replicated, there is no reason to hold on to the claim as true.

### 1.4 Poor replications of *P*

Some recent studies have stirred up the debate about a scientific replication crisis. In a large multicenter study, 100 seemingly well‐performed experimental and correlational studies in the field of psychology were reperformed similarly as they were reported in the original publications.^23^ The aim of the study was to investigate the rate of successful replication. There is no single standard for concluding when a test is successfully replicated, but among the different approaches tested was an investigation into whether a significant original test result (*P* < 0.05) turned out significant in the study replication also.

Of the original studies, 97% had *P*‐values lower than 0.05. In the replicated studies, only 36% had *P*‐values lower than 0.05. The average study replication power was 0.92, indicating that if the originally detected effects were true and accurately measured; approximately 89 of the 97 (92%) studies would have reached statistical significance in the replication studies also.

It is likely that at least some of the original study findings were wrong (false positive results), and it is also likely that some of the replicated studies failed to find an existing difference or correlation (false negative results). But a statistically significant replication rate of 36% is by any means low.

Although the *P*‐values in the original studies were all low (likely an informal prerequisite for publication), this was not the case for the replicated *P*‐values, which were widely distributed between 0 and 1.^23^ Such a distribution suggests that original study type 1 errors are more likely the reasons for the low replication rate than replication study type 2 errors. In other words, low *P*‐values in psychological research seem to be unreliable measures of supporting evidence of the tested hypotheses. This also seems to be true in laboratory economic research^24^ and in typical experimental study group settings.^25^ No large‐scale replication studies have yet, to the best knowledge, been performed in the field of clinical medicine, but if ever done, a poor replication of low *P*‐values should come as a no surprise.

### 1.5 Reasons for falsely low *P*

To further understand why *P*‐values from seemingly good research may be unreliable as evidential measures, one needs to understand that *P*‐values end up low for different reasons. A *P*‐value may indeed be low because the null hypothesis is false, but it may also turn out low when the null hypothesis is true. The latter occurs when there is no real difference (or correlation) between compared groups, but random or systematic errors provided the researcher with extreme data. The risk of getting low *P*‐values due to random errors is inflated by nonsystematic exploration, repeated testing, and data flexibility (where researchers tend to make decision about the collection and analysis of data, which coincides with their desires)^26^ and systematic errors may occur due to several and different reasons, including measurement errors, observer bias, placebo effects, and parameter changes that occur over time. The risk of having unusual data due to either random or systematic errors is not formally estimated within the test procedure and is rarely reflected upon in scientific publications. The risk of unwillingly conducting such errors may be subtle even for researchers adhering to stringent research methodology and predefined analysis plans. In addition to that, in the absence of study protocols predefining study outcomes, false positive *P*‐values may be inflated in the published literature due to selective reporting^27^ as well as through the multiple factors believed to contribute to publication bias.^14^

### 1.6 So where does this leave *P*?

Null hypothesis significance testing has been around a long time and will most likely continue to remain an important method in quantitative analysis. It is, however, time to acknowledge that solitary *P*‐values themselves do not provide reliable evidence for or against any hypothesis, despite being lower than, say, *P *< 0.001.

A *P*‐value without context or other scientific reasoning provides limited information. A low *P*‐value from a test must be weighed against the study's question, design, and conduct, including a thorough understanding of the data collection, management, and number of tests performed. It has been suggested that *P*‐values may be supplemented or replaced emphasizing parameter and interval estimation allowing for sampling uncertainty,^13^ Bayesian inferential methods,^5^ as well as alternative measures of evidence, such as likelihood ratios^28^ preferably allowing conscientious statements about both the precision and plausibility in the conclusions.

Another suggested approach is to use prediction markets to estimate the reproducibility of scientific research findings or to assess a probability of a hypothesis being true.^29^ As pointed out in a statement by the American Statistical Association warning over the misuse of *P*‐values, such complementing methods may more directly address the size of an effect or whether a hypothesis is correct.^28^

Although it is not possible, with certainty, to know whether a result of a statistical analysis represents the truth or not, different frameworks have been proposed to model the amount of false positive findings. Such models may depict the long run perspective presenting a positive predictive value^22^ as well as models how to interpret a single *P*‐value.^18^ Both these approaches imply that low *P*‐values may not be the solid evidential measures most believe them to be.

## CONFLICTS OF INTEREST

The author has no conflicts of interest to report.
